# Supplementary material for: Oscillatory brain activity in spontaneous and induced sleep stages in flies
Source: Nat Commun. 2017 Nov 28;8:1815. doi: 10.1038/s41467-017-02024-y (PMC5704022; doi:10.1038/s41467-017-02024-y)
Supplement: Supplementary file 1 — Supplementary Information [file 41467_2017_2024_MOESM1_ESM.pdf]

Fig. S1

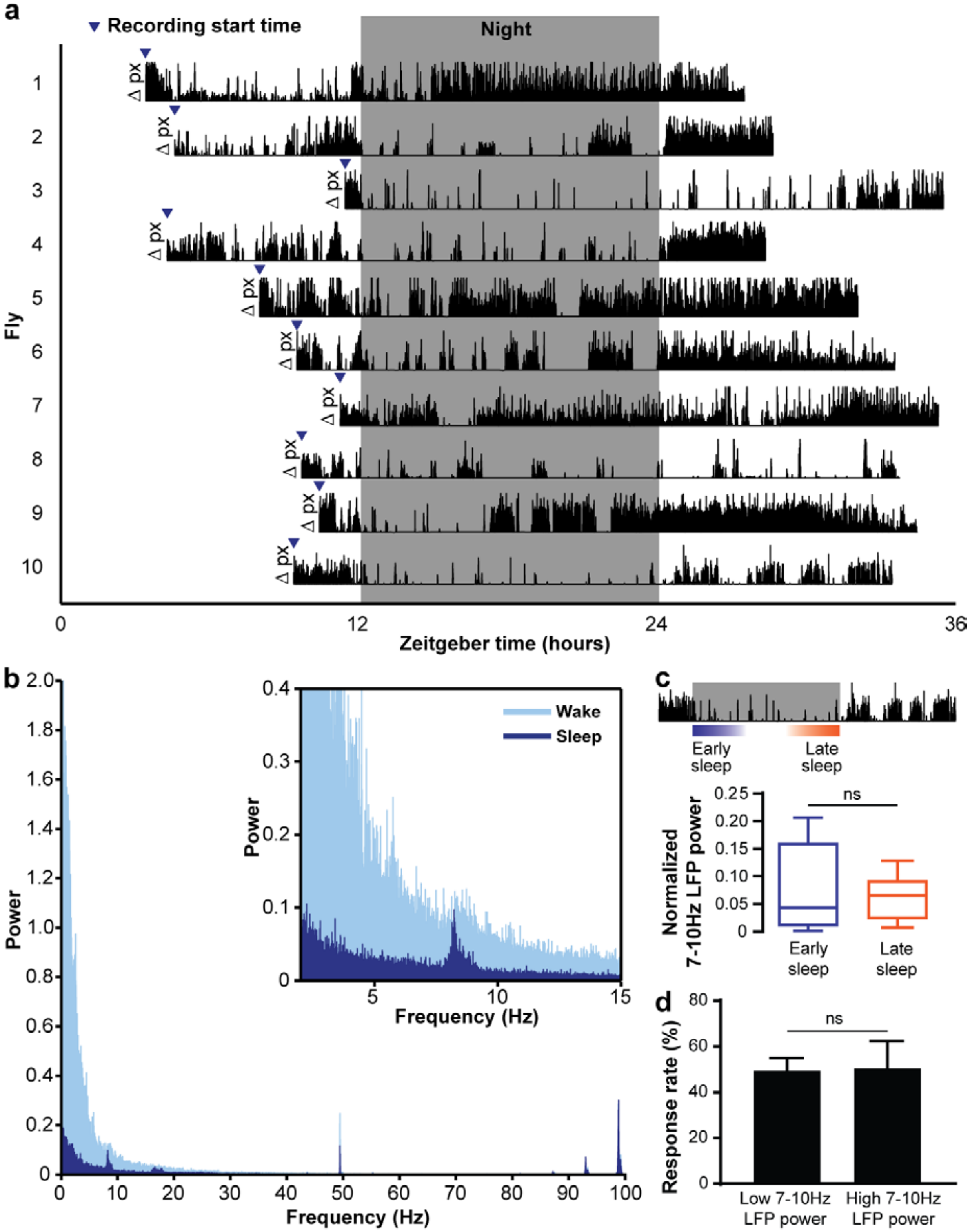

Supplementary Figure 1. Oscillatory brain activity during fly sleep. (a) 24-hour movement traces for flies recorded on the overnight setup. Recording start time varies across flies (blue arrow) and all recordings included the entire 12-hour night period (gray shade). (b) Comparison of LFP power for a sample fly between the entire wake and sleep period (>5 min inactivity) for frequencies between 0 and 100Hz. Inset shows a close-up view on frequencies between 2 and 15Hz. (c) Normalized median LFP power for the 7-10Hz domain of the first third of night sleep compared to the last third of night sleep (schema in top panel). No significant difference between early night sleep 7-10Hz oscillation amplitudes compared to the late sleep ones (n=10, p=0.9219, Wilcoxon matched-pairs signed rank test, two-tailed). (d) No significant difference in behavioral responsiveness was detected when 7-10Hz power during sleep was high, compared to when it was low (n=6, p=0.5625, Wilcoxon matched-pairs signed rank test, two-tailed).

Fig. S2

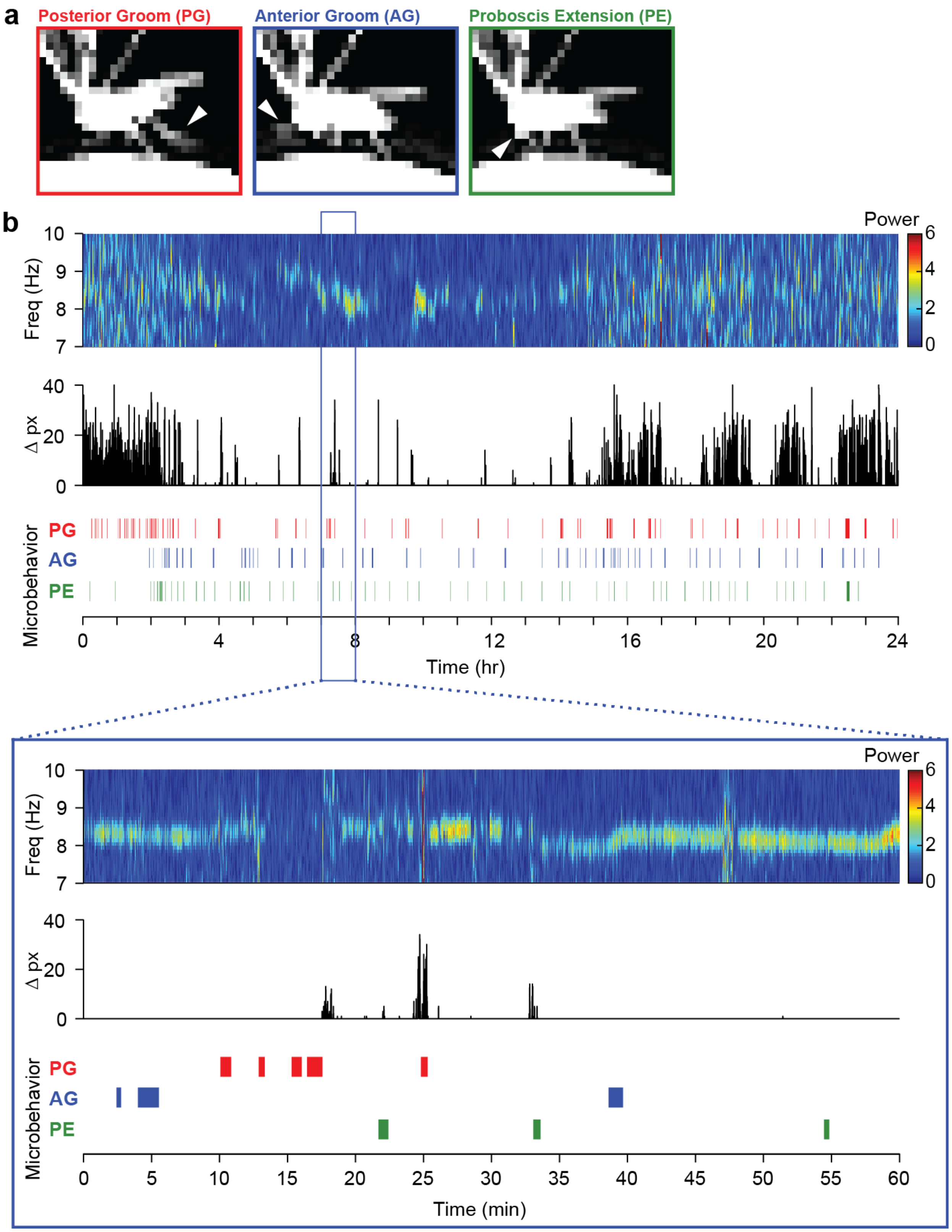

Supplementary Figure 2. Oscillations not associated with microbehaviors. (a) Screenshots of movie recordings showing the three categories of fly microbehaviors observed: Posterior Groom (PG, red, left panel), Anterior Groom (AG, blue, middle panel), and Proboscis Extension (PE, green, right panel). White arrows indicate the animated region of the movie recording observed for the respective microbehaviour. (b) Top half shows a 24-hour recording of a fly, displaying LFP power in the frequency range of 7-10Hz (top), with the corresponding movement trace (middle), and time of occurrence for each of the three microbehaviors (bottom). Bottom half shows the close-up spectrogram, movement traces, and microbehaviors occurrence at the 7th to the 8th hour mark. Images: Melvyn Yap.

Fig. S3

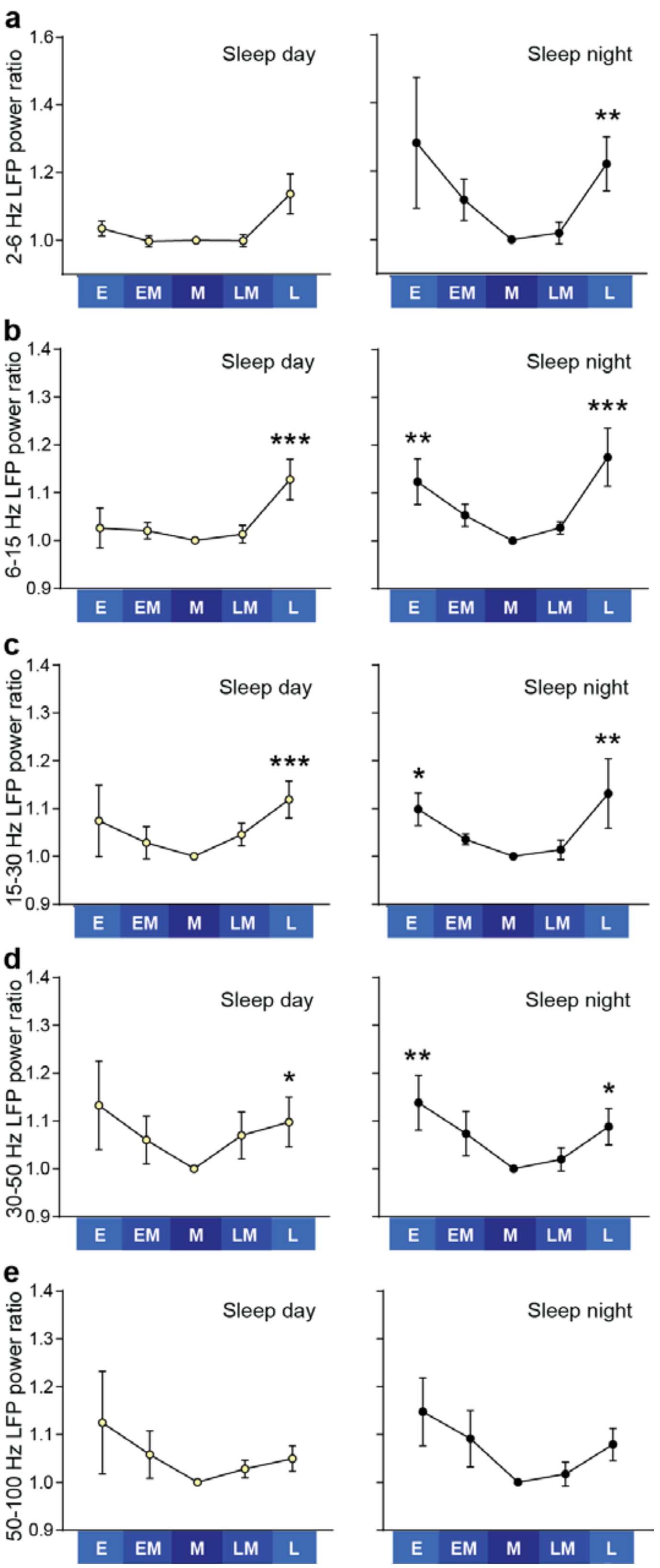

Supplementary Figure 3. Sleep stages. (a) Average 2-6Hz power (±s.e.m.) for each sleep epoch, normalized to mid-sleep power. (b) Average 6-15Hz power (±s.e.m.) for each sleep epoch, normalized to mid-sleep power. (c) Average 15-30Hz power (±s.e.m.) for each sleep epoch, normalized to mid-sleep power. (d) Average 30-50Hz power (±s.e.m.) for each sleep epoch, normalized to mid-sleep power. (e) Average 50-100Hz power (±s.e.m.) for each sleep epoch, normalized to mid-sleep power (for A-E, n = 10, *p < 0.05, **p < 0.01, ***p < 0.001 by Friedman test with Dunn's multiple comparisons between each sleep segment and mid-sleep). Sleep epochs are the same as described in Fig. 1f.

Fig. S4

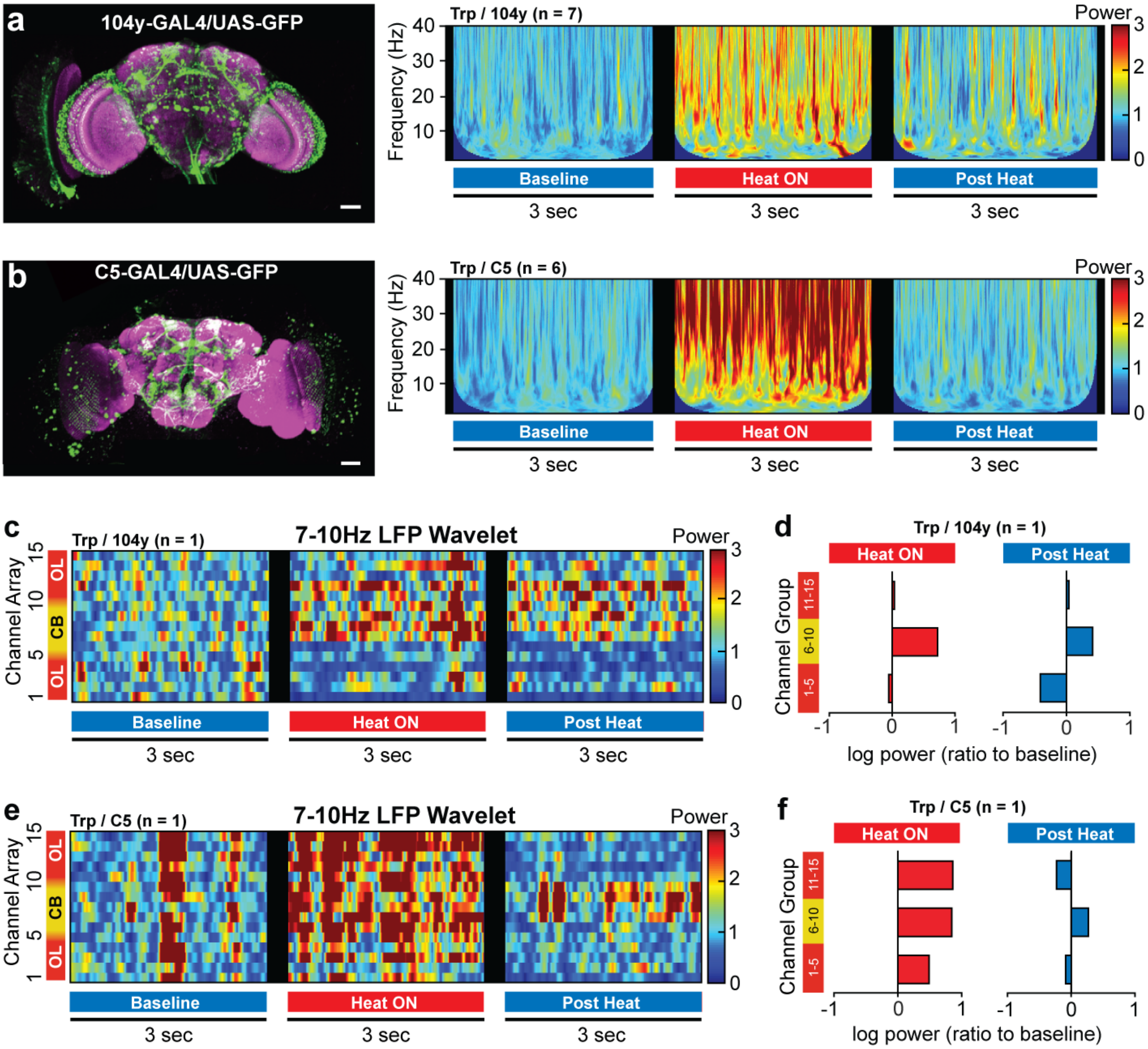

Supplementary Figure 4. Multichannel recordings. (a) Expression pattern of 104y-Gal4 circuit (green) as visualized by green fluorescent protein (GFP) expression (left). Scale bar = 10 µm. Averaged spectrogram of 104y-Gal4/UAS-TrpA1flies (n=7) for the central brain channels (channel 6-10), for the 2-40Hz frequency range (right). Red bar indicates circuit activation time. Black vertical bars represent excluded data; 3s epochs were not immediately contiguous. (b) Expression pattern of c5-Gal4 circuit (green) as visualized by green fluorescent protein (GFP) expression (left). Scale bar = 10 µm. Averaged spectrogram of c5-Gal4/UAS-TrpA1flies (n=6) for the central brain channels (channel 6-10), for the 2-40Hz frequency range (right). Black vertical bars represent excluded data; 3s epochs were not immediately contiguous. (c) Individual 7-10Hz spectrogram for a 104y-Gal4/UAS-TrpA1 experiment. (d) Average 7-10Hz activity for data shown in c, during (red) and after heat (blue) for the three brain regions, as in Fig. 2c. (e) Individual 7-10Hz spectrogram for a c5-Gal4/UAS-TrpA1 experiment. (f) Average 7-10Hz activity for data shown in e, during (red) and after heat (blue) for the three brain regions, as in Fig. 2c. Images: Angelique Paulk.

Fig. S5

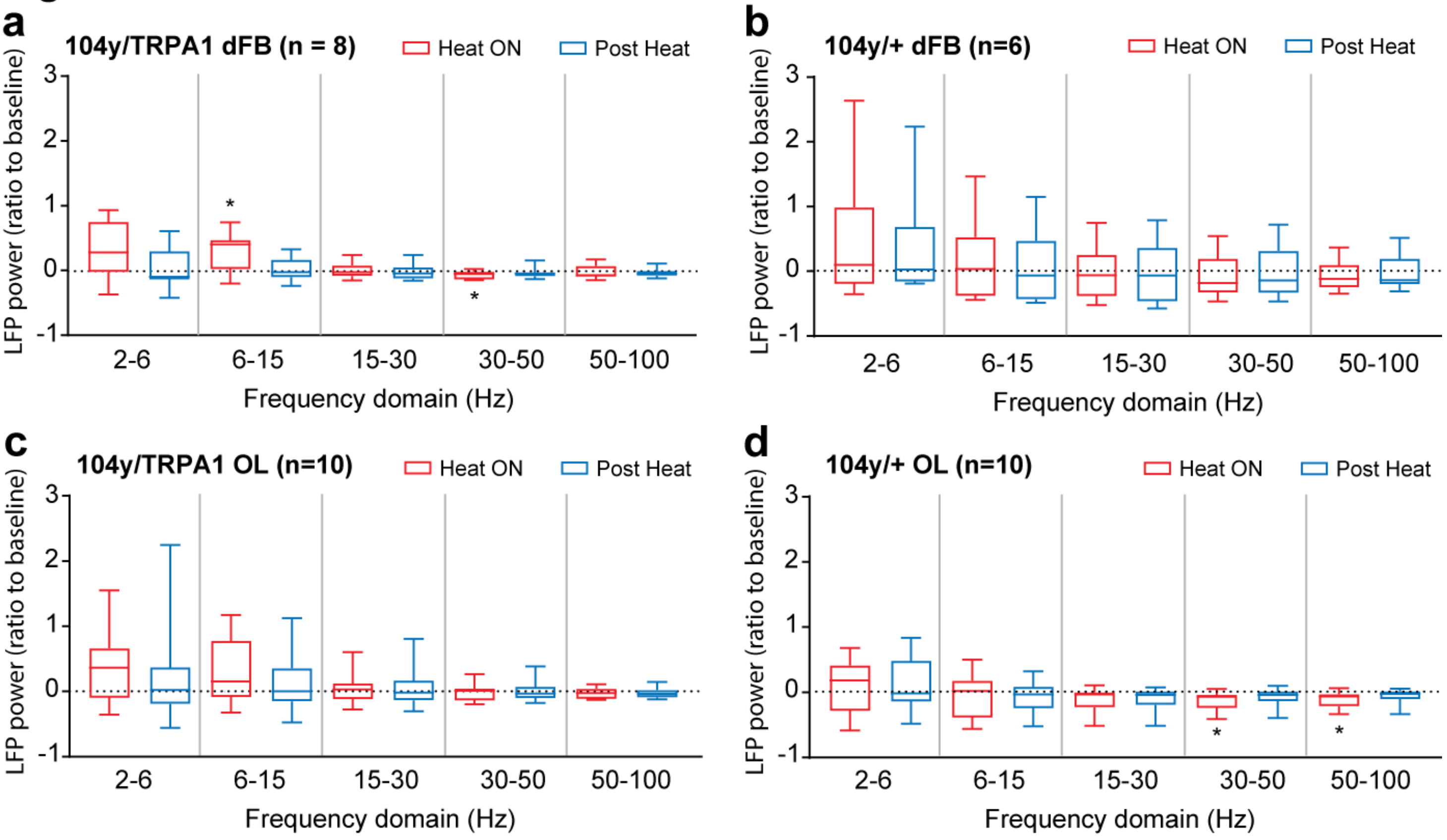

Supplementary Figure 5. Dorsal fan-shaped body recordings. (a) Increase in 6-15Hz LFP power in the dorsal fan-shaped body (dFB) during Heat ON was statistically significant for TRPA1/104y flies, with a significant decrease in 30-50Hz LFP power also observed (n = 8, *$p < 0.05$ by one sample t-test comparing to baseline of zero). No significant change in LFP power was observed for all other conditions (ns, by one sample t-test comparing to baseline of zero for all except Post Heat: 30-50Hz, ns, by Wilcoxon signed rank test). (b) LFP power in the dFB remain unchanged for all frequency domains for +/104y flies (n = 6, ns by one sample t-test comparing to baseline of zero for all except Post Heat and Heat ON: 2-6Hz, ns, by Wilcoxon signed rank test). (c) No significant increase in LFP power in the optic lobe (OL) during Heat ON across all frequency domains for TRPA1/104y flies (n = 10, ns by one sample t-test comparing to baseline of zero for all except Post Heat: 2-6Hz, 6-15Hz, 15-30Hz, ns, by Wilcoxon signed rank test). (d) Significant decrease in 30-50Hz and 50-100Hz LFP power in the OL for +/104y flies (n = 10, *$p < 0.05$, by one sample t-test comparing to baseline of zero). No significant change in LFP power was observed for all other conditions (ns, by one sample t-test comparing to baseline of zero for all except Heat ON: 15-30Hz, Post Heat: 15-30Hz, 50-100Hz, ns, by Wilcoxon signed rank test).

Fig. S6

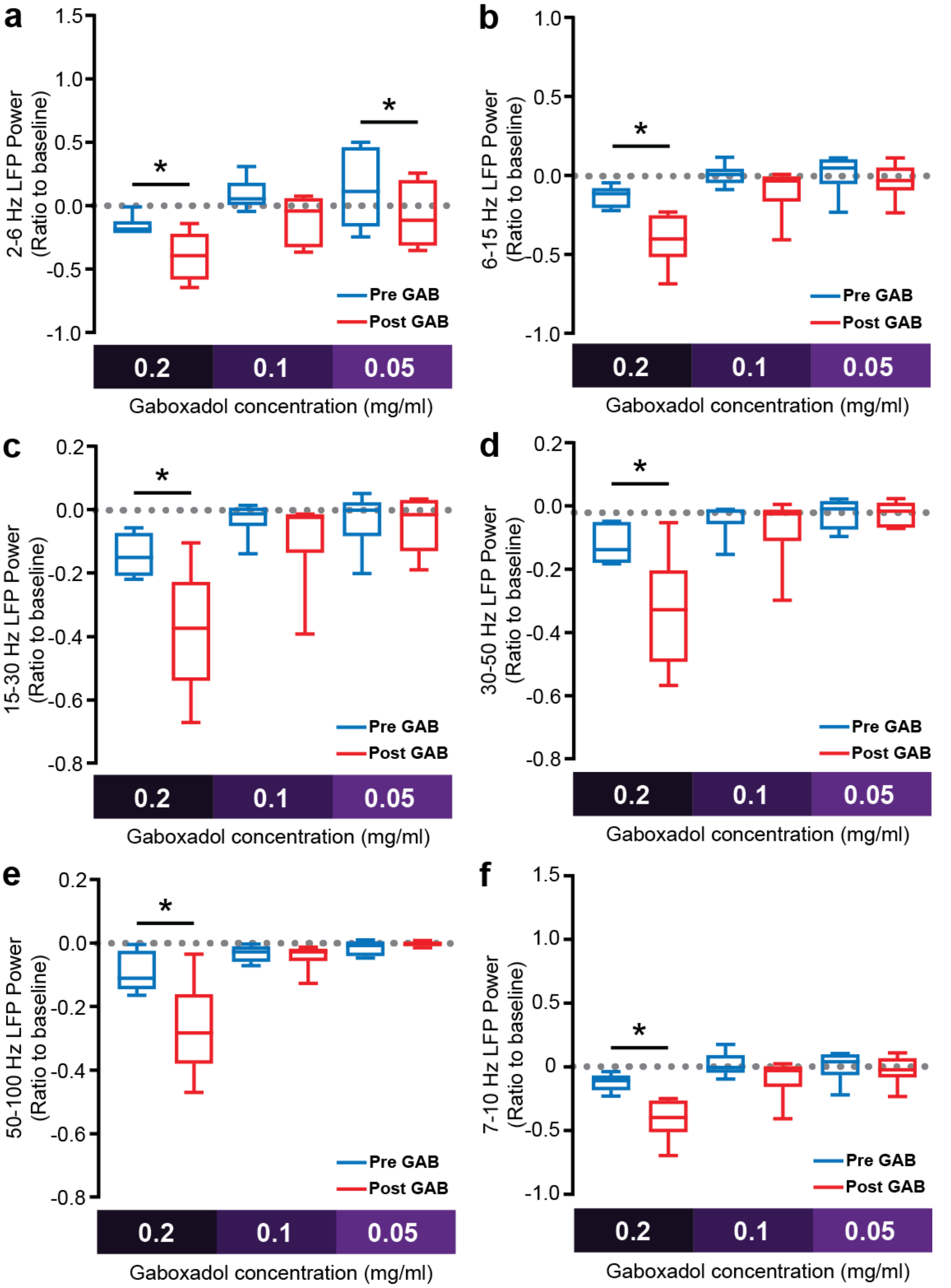

Supplementary Figure 6. Pharmacologically-induced sleep. (a) A significant decrease in the 2-6Hz LFP power was observed when flies were exposed to Gaboxadol 0.2mg/ml (n = 6) and 0.05mg/ml (n = 6). (b) A significant decrease in the 6-15Hz LFP power was observed when flies were exposed to Gaboxadol 0.2mg/ml but not for lower concentrations. (c) A significant decrease in the 15-30Hz LFP power was observed when flies were exposed to Gaboxadol 0.2mg/ml but not for lower concentrations. (d) A significant decrease in the 30-50Hz LFP power was observed when flies were exposed to 0.2mg/ml but not for lower concentrations. (e) A significant decrease in the 50-100Hz LFP power was observed when flies were exposed to Gaboxadol 0.2mg/ml but not for lower concentrations. (f) A significant decrease in the 7-10Hz LFP power was observed when flies were exposed to Gaboxadol 0.2mg/ml but not for lower concentrations (for all frequency domains tested, *$p < 0.05$ by Wilcoxon matched pairs signed rank test between pre and post drug).

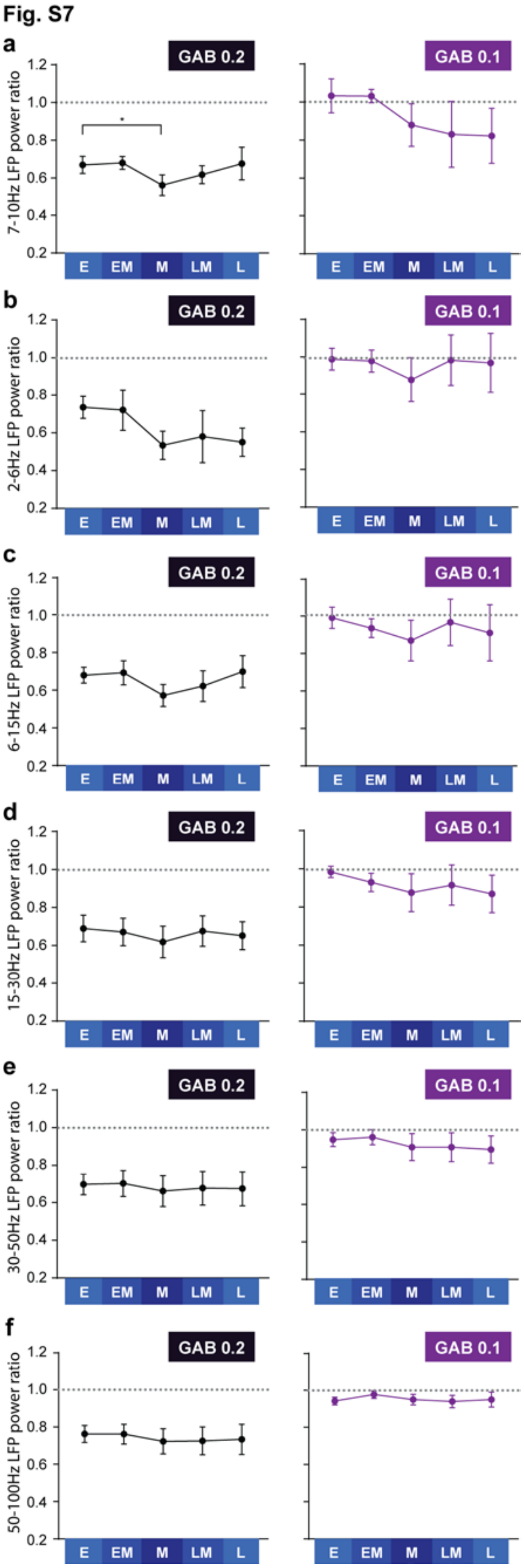

Supplementary Figure 7. Sleep stages. (a) Average (±s.e.m.) power for Gaboxadol-induced sleep partitioned into five sleep segments (as in Fig. 1f), normalized to baseline (first 5 min of recording) for frequency domain 7-10Hz, (b) 2-6Hz, (c) 6-15Hz, (d) 15-30Hz, (e) 30-50Hz, (f) 50-100Hz (for a-f, n = 6 for 0.2mg/ml Gaboxadol, n = 5 for 0.1mg/ml Gaboxadol, *$p < 0.05$ by Friedman test with Dunn's multiple comparisons between each sleep segment). E, early sleep; EM, early-mid sleep; M, mid sleep; LM, late-mid sleep; L, late sleep.

Fig. S8

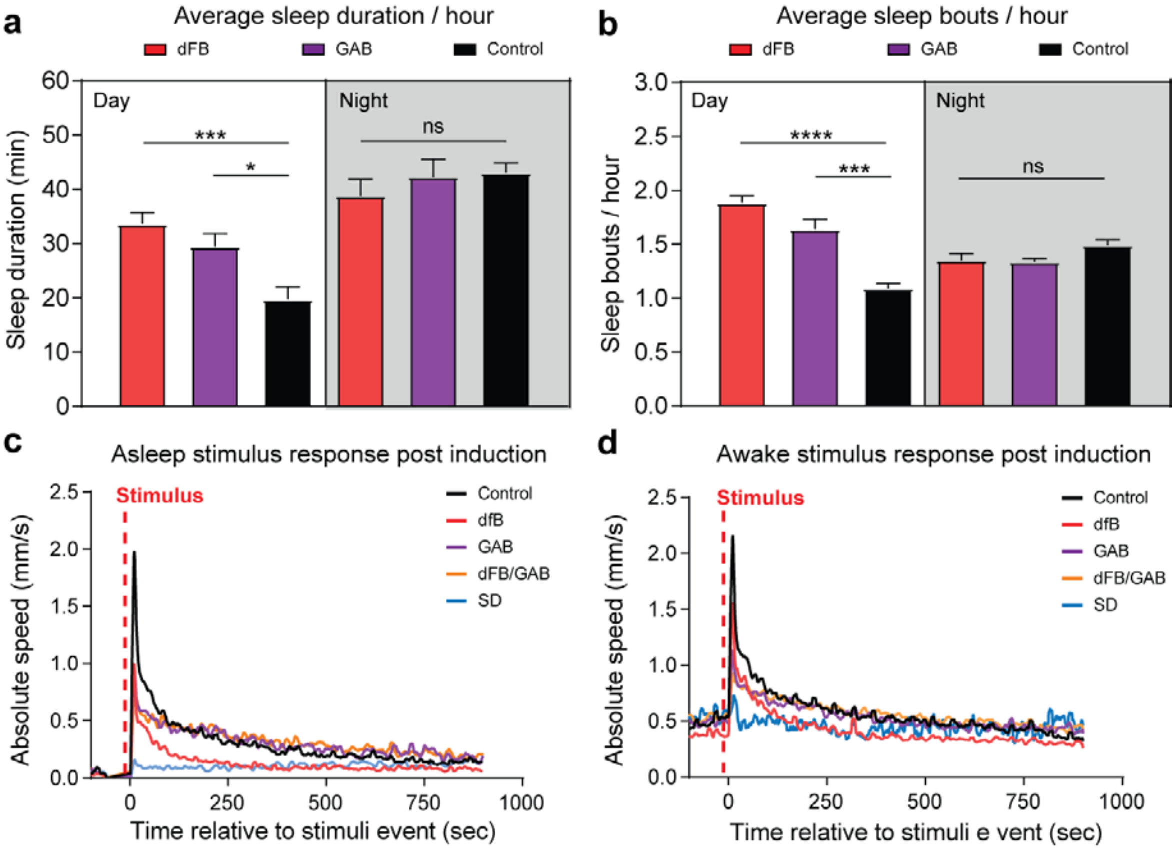

Supplementary Figure 8. Behavioral effects of induced sleep. (a) Average sleep duration (±s.e.m.) for flies induced to sleep for 24 hours by either optogenetic dFB activation (dFB) or Gaboxadol (GAB), compared to controls (n= 102 for each group). Both dFB and GAB groups had significantly increased daytime sleep compared to the controls. *p < 0.05, ***p < 0.01 (ANOVA with Tukey's test for multiple comparisons). (b) Average number of day and night sleep bouts per hour (±s.e.m). ***p < 0.001, ****p < 0.001 (ANOVA with Tukey's test for multiple comparisons). (c) Responsiveness of sleeping flies to stimuli, evaluated by the fitted peak responsiveness amplitude. The sleeping controls had a higher peak responsiveness to stimuli ($F(4, 505) = 17.77$, dFB vs Control, p = 0.0009, GAB vs Control, p = 0.0001, dFB/THIP vs Control, p < 0.0001. There was no significant different in peak responsiveness between dFB vs GAB, p = 0.99, or GAB vs dFB/THIP, p = 0.98, or dFB vs dFB/THIP, p = 0.93. All conditions had increased peak amplitude responsiveness compared to sleep-deprived flies, SD vs Control, p <0.0001, SD vs dFB, p = 0.0001, SD vs GAB, p = 0.0007, SD vs dFB/THIP, p = 0.002. The stimulus time is indicated by the red dashed line. (d) Peak responsiveness to stimuli in awake flies, represented by the average difference between the pre-and post-stimuli speeds of awake flies (*i.e.*, moving in the past 4min) and evaluated by the fitted peak responsiveness amplitude. There was no significant difference between conditions ($F(4, 505) = 3.79$, dFB vs Control, p = 0.93, GAB vs Control, p = 0.29, dFB/GAB vs Control, p = 0.16, dFB vs GAB, p = 0.77, GAB vs dFB/GAB, p = 0.99, dFB vs dFB/GAB, p = 0.58, GAB vs SD, p = 0.47, dFB/GAB vs SD, p =0.67. Controls and dFB flies had increased responsiveness compared to sleep deprived flies, SD vs Control, p = 0.003, SD vs dFB, p = 0.04. Significance was determined by ANOVA with Tukey's test for multiple comparisons. Data in c and d are from the same flies as in Fig. 6e and f.

Supplementary Note 1

Multichannel recordings 2-40Hz analysis

We report the results of the Aligned-Rank ANOVA from an Analysis of Deviance Table using Type III Wald F tests with Kenward-Roger degrees of freedom. For the 2-40Hz power analysis, there was a significant three-way-interaction for FlyGroup x Condition x Region ($F = 2.9974$, $df = 144$, $p < 0.01$) for the 104y line. To test interactions we Bonferroni corrected a 0.05 alpha value depending on the number of comparisons. Post-hoc contrast indicated there is a statistically significant increase in central brain activity relative to the optic lobes for the 104y-TRP line during heat compared to baseline (Chi square = 17.04, $p < 0.0125$). The comparison to heat off was also significant ($F = 7.9711$, $p < 0.0125$). The same post-hoc contrast was performed between the 104y-TRP line and UAS-TRP control and had non-significant increase during heat on and a non-significant decrease during heat off ($p > 0.1$).

The C5 line also had a statistically significant 3-way interaction ($F = 4.2575$, $df = 136$, $p < 0.001$). The contrast comparing the center to both optic lobes was non-significant both for heat on (Chi square = 3.1372, $p = 0.07652$) and heat off (Chi square = 0.3391, $p = 0.5603$) for the C5-TRP versus GAL4 comparison. However, this line showed increased activity in one of the optic lobes as well as the center, so we did another contrast comparing the center and optic lobe to the other (contrast scheme used: -1, 1/2, 1/2). There was a significant increase for the heat on condition (Chi square = 7.5004, $p < 0.00625$), but not heat off (Chi square = 0.3985, $p = 0.5279$). Finally, we looked at changes at the single optic lobe relative to the center and other optic lobe (contrast scheme used: -1/2, -1/2, 1) and found a statistically significant increase during heat on (Chi square = 20.339, $p < 0.00625$) but not heat off (Chi square = 1.4729, $p = 0.2249$). We did this same comparison for a single optic lobe for the C5-TRP line compared to UAS-TRP control and found a significant increase (Chi square = 16.334, $p < 0.00625$) for heat on with a Bonferroni corrected alpha cut-off of 0.00625, but not for heat off (Chi square = 1.5028, $p = 0.2202$). What this indicates is that there is a broad increase in 2-40Hz activity during heating for the c5-Gal4/UAS-TrpA1 line compared to the Gal4 control, across both optic lobes and the central brain, and that this activity is most prominent in one of the optic lobes. This pattern of activity biased to the optic lobe is not present in the UAS-TrpA1 control. We also did an analysis investigating these same contrasts for the frequency band clusters[21], excluding 0-2Hz, summarized in Supplementary Table 1 and 2.

Frequency x time analysis

The final analysis concerned the frequency x time plots for the 2-15Hz frequency band, organized into 40 frequency bins. As before, an Aligned-Rank ANOVA was performed on this dataset which had been log transformed. A three-way interaction was investigated with the form FlyGroup x Condition x FreqBand, where FreqBand has 40 levels. The 104y group had a significant interaction between FlyGroup and Condition ($F = 111.80075$, $df = 1428$, $p < 0.001$), but no significant three-way interaction. The same goes for C5, where FlyGroup x Condition was significant ($F = 105.97697$, $df = 1309$, $p < 0.001$), but C5 also had a significant interaction with FlyGroup x FreqBand ($F = 2.64535$, $df = 1309$, $p < 0.001$). As recommended by Wobbrock (2011), we redid the aligned rank procedure for the 2-way interaction term of interest before checking contrasts. We sought to investigate each of these significant terms, with FlyGroup and Condition coded as earlier, but FreqBand coded to investigate the contribution of frequencies between 7.3-10Hz relative to all other frequencies set equal (2-6.9Hz, 10.3-15Hz).

The FlyGroup x Condition comparison with Heat On being compared to Baseline was statistically significant (Chi square = 266.6, $p < 0.001$), as was Post Heat (Chi square = 114.47, $p < 0.001$). For C5, Heat On was significant (Chi Square = 191.97, $p < 0.001$), but not Post Heat (Chi square = 0.0483, $p = 0.826$). For C5, the FlyGroup x FreqBand comparison was non-significant (Chi square = 0.3507, $p = 0.5537$), indicating no specific 7-10Hz band activity could be detected.

A note on statistical analyses for the multichannel data

Rank-based ANOVAs generally only differ from parametric ANOVA or regression methods in their manner of rank-transforming data [1, 2]. The most popular of these convert response data into ordinal ranks from 1 to N [1, 3], however, as this ordinal rank method fails to adequately cope with interaction effects, the Aligned-Rank procedure was developed [4], which we use here. As the name suggests, the Aligned-Rank transformation uses a mixture of response ranking and alignment [5]. Use of the Aligned-Rank transformation makes the assumption that main effects are nuisance effects and aligns data in terms of their interaction terms before rank transforming them into percentiles. The Aligned-Rank ANOVA has been demonstrated to have more power to

detect differences between multi-factor groups for non-parametric data compared to parametric techniques, particularly when extreme skew, non-orthogonal design, or violations of normality are present [6].

Supplementary Table 1

Summary of Analysis of Variance of Aligned Rank Transformed Data. The figure contains a summary of p-values from an Analysis of Deviance Table (Type III Wald F tests with Kenward-Roger df), a mixed model tested (lmer) on the ranked transformed data. The model being tested in every case is: Response ~ FlyGroup x Condition x Region + (1|Fly), where (1|Fly) indicates a random slope being fitted to account for within-subject variation and Response is the averaged power for a given frequency band. *C5 2-6Hz had a significant two-way interaction FlyGroup*Region significant (p < 0.01).

**FlyGroup x Condition x Region**	**2-6Hz**	**6-15Hz**	**15-30Hz**	**30-50Hz**	**50-100Hz**
104y	<0.001	<0.001	**<0.01**	**<0.05**	**<0.05**
C5	ns*	<0.001	<0.001	<0.001	**<0.001**

Supplementary Table 2

Results of Three-Way Helmert Contrasts. P-values are from the result of a Chi-Square test conducted using the phia R package on the Aligned Rank model. For all cases the heat on condition had a significant increase in the center across all frequency bands but differed in the region for each fly line. The C5 flies only had a non-significant increase for one of the optic lobes (11-15 channels) and the center, relative to the other optic lobe, while the 104y line had a significant increase only in the center relative to the optic lobes. In all cases except 104y 15-30Hz and 30-50Hz the heat off contrast was non-significant. [1] C5 2-6Hz only had a significant interaction between FlyGroup and Region, so two comparisons were made, differing only in how Region was coded (Bonferroni corrected alpha = 0.025). First, the center was tested to the optic lobes and was non-significant (Chi square = 2.1877, p = 0.1319). [2] Then channel group 11-15 and center was compared relative to channel group 1-5 and had a non-significant increase (Chi square = 3.3435, p = 0.06747).

**Condition Contrast Term**	**2-6Hz**	**6-15Hz**	**15-30Hz**	**30-50Hz**	**50-100Hz**
Heat On vs Baseline (104y) alpha = 0.025	**<0.001**	**<0.001**	**<0.001**	**<0.001**	**<0.001**
Heat Off vs Baseline (104y) alpha = 0.025)	ns (0.03546)	ns (0.04502)	<0.05	<0.025	ns (0.02556)
Heat On vs Baseline (C5) alpha = 0.0125	[1]	ns (0.03221)	**< 0.001**	**< 0.001**	**< 0.001**
Heat Off vs Baseline (C5) Alpha = 0.0125	[2]	ns (0.9196)	ns (0.4524)	ns (0.123)	ns (0.2333)

Supplementary References

1. Conover, W.J. & Iman, R.L. Rank transformations as a bridge between parametric and nonparametric statistics. *The American Statistician* **35**, 124-129 (1981).
2. Zimmerman, D.W. & Zumbo, B.D. Relative power of the Wilcoxon test, the Friedman test, and repeated-measures ANOVA on ranks. *The Journal of Experimental Education* **62**, 75-86 (1993).
3. McKean, J.W. & Hettmansperger, T.P. Tests of hypotheses based on ranks in the general linear model. *Communications in statistics-theory and methods* **5**, 693-709 (1976).
4. Salter, K. & Fawcett, R. A robust and powerful rank test of treatment effects in balanced incomplete block designs. *Communications in Statistics-Simulation and Computation* **14**, 807-828 (1985).
5. Hodges, J. & Lehmann, E.L. Rank methods for combination of independent experiments in analysis of variance. *The Annals of Mathematical Statistics* **33**, 482-497 (1962).
6. Beasley, T.M. Multivariate Aligned Rank Test for Interactions in Multiple Group Repeated Measures Designs. *Multivariate Behavioral Research* **37**, 197-226 (2002).
