# Supplementary material for: Oscillatory brain activity in spontaneous and induced sleep stages in flies
Source: Nat Commun. 2017 Nov 28;8:1815. doi: 10.1038/s41467-017-02024-y (PMC5704022; doi:10.1038/s41467-017-02024-y)
Supplement: Supplementary file 3 — Description of Additional Supplementary Information [file 41467_2017_2024_MOESM3_ESM.pdf]

Description of Additional Supplementary Files

File Name: Supplementary Movie 1

Description: The sleep induction effect of TrpA1 activation of the dorsal fan-shaped body neurons (TRPA1/104y) is evident by a decrease in movement (top left panel) when the brain was perfused with heated solution, associated with an increase in 7-10Hz power in the local field potential (bottom left panel). This effect was not observed in the control strain (104y/+; right panels). Movie playback at 15x the normal speed.
